# Infectious reservoir of *Plasmodium *infection in Mae Hong Son Province, north-west Thailand

**DOI:** 10.1186/1475-2875-3-34

**Published:** 2004-09-22

**Authors:** Aree Pethleart, Somsak Prajakwong, Wannapa Suwonkerd, Boontawee Corthong, Roger Webber, Christopher Curtis

**Affiliations:** 1 London School of Hygiene and Tropical Medicine, London, WC1E 7HT, UK; 2 Faculty of Medicine, Thammasat University, Pathumtani 12120, Thailand; 3 Vector Borne Disease Section, Office of Disease Prevention and Control No.10, Chiang Mai, 52000, Thailand; 4 Vector-borne Disease Control Unit No.8, Mae Hong Son Province, Thailand

## Abstract

**Background:**

It was unknown whether the main reservoir of *Plasmodium falciparum *and *Plasmodium vivax, *which infects mosquitoes in Thailand, was (a) in people feeling sufficiently ill with malaria to come to a clinic or (b) in people who had remained in their home villages with some fever symptoms or with none.

**Methods:**

Mass surveys were carried out in Thai villages to identify people with *Plasmodium *infections and with fever. Malaria patients were also located at a clinic which served these villages. Adults from both sources whose blood slides registered positive for *Plasmodium *spp. were requested to allow laboratory-bred *Anopheles minimus *to feed on them. Seven to nine days after the blood feeds the mosquitoes were dissected and checked for presence of oocysts.

**Results and Discussion:**

There were higher rates of *Plasmodium *infection among people in the villages with fever than without fever and much higher rates of infection among clinic patients than among people who had remained in the villages. People with malarial infections identified via the clinic and the village surveys could infect mosquitoes, especially, but not only, if their blood slides showed visible gametocytes. Because only a very small minority of the village populations were visiting the clinic on any one day, assessment indicated that the main reservoir of infection was not primarily among clinic patients but among those in the villages, especially those feeling feverish.

**Conclusions:**

Efficient use of an anti-gametocyte drug to suppress the parasite reservoir in a population requires that it be given, not just to clinic patients, but to infected people located by mass surveys of the villages, especially those feeling feverish.

## Introduction

An estimate of the infectiousness of the *Plasmodium* reservoir to mosquitoes is of interest in understanding the epidemiology of malaria and its changes after application of certain types of control measures. Different approaches have been used to investigate this, including direct feeding of mosquitoes through the skin of human subjects [1-5] or feeding through an artificial membrane [6-13]. The present study was designed to determine whether the reservoir of infection of vectors was mainly in people ill enough to go to a clinic or whether it was mainly in those with slight or no malarial symptoms who have remained in their villages.

## Patients and Methods

### Subjects

This study was approved by the Thai Ethics Committee and the Ethics Committee of London School of Hygiene and Tropical Medicine. Subjects were recruited from mass blood surveys in villages and from a clinic in Muang District, Mae Hong Son Province, which is in north-western Thailand. Anti-malarial drug use is tightly controlled in Thailand and these drugs are generally available on prescription. Self-medication is, therefore, unusual in this area. Mass blood surveys were conducted twice a year. After slides were examined for parasites, the people whose slides were positive and were more than 14 years of age were invited to take part in this study. The Thai ethical committee did not allow direct feeding of mosquitoes on children aged less than 15 years. Adults positive for malaria parasites were informed about the purpose of the study and, if they agreed, a consent form was signed and patients were interviewed.

Human subjects for the study were also enrolled from the Vector-borne Disease Control Unit No. 8 (VBDU: described as "Clinic" throughout this paper). In this Clinic, blood smears were taken by the clinic staff and stained with Giemsa to check for malaria. Patients who had presented with clinical malaria and/or parasitaemia and were more than 14 years of age were informed about the purpose of the study and asked to participate in it. If they agreed and signed the consent form, then the mosquito feeding was performed.

### Mosquito feeding and dissection

Before feeding, patients' arms were cleaned with 70% alcohol. Then, 50 laboratory-bred female *Anopheles minimus *species A (aged 4–6 days), which had been starved for 9–12 hr, were allowed to feed on their arms for 30 minutes. After feeding was completed, anti-malarial drugs were given to all the human subjects by a malaria worker. Two hours after feeding the unengorged mosquitoes were removed from the cups using a sucking tube and destroyed, leaving only fully engorged mosquitoes in the cup. The mosquitoes were brought back to the Chiang Mai insectarium and maintained at 25 to 27 C and 70–80% relative humidity with permanent access to sucrose solution and without any further blood meals. Mosquitoes at 7–9 days post-feed were anaesthetized and the wings and legs were removed. Midguts were dissected on glass slides in a drop of 0.85% NaCl and examined at a 40× magnification. The number of oocysts present on the midgut to each mosquito was counted and recorded individually.

### Quality control of blood slide data

Asexual parasitaemia and gametocytaemia were quantified as the number of aseuxal forms/200 white blood cells on a thick film. Throughout the period of the study, 20% of the negative blood smears and all positive slides which had been examined by the microscopist of the Clinic were chosen randomly and re-examined by a team from the Office of Vector-borne Disease Control No.2, Chiang Mai. All cases of positive slides without visible gametocytes and which led to oocyst production were re-examined by an expert team from the London School of Hygiene and Tropical Medicine to ascertain whether a few gametocytes might have been present but were initially missed.

### Analyses

The database from mosquito feeding included 1) densities of gametocytes and trophozoites, 2) number of infected mosquitoes, and 3) mean number of oocysts per infected mosquito. The chi-square test or Fisher's exact test was used to examine the significance of differences in the proportion of people in various categories who transmitted malaria to the mosquitoes (i.e. yielded at least 1 oocyst). Regressions on the natural log of gametocyte density were also computed. Stata statistical software (version 6) was used for analysis.

## Results

The upper part of Table 1 shows the results associated with 5,227 blood smears from children and adults in village surveys. 10.7% (561/5,227) of subjects reported fever within the previous seven days but the corresponding rate for slide positives was much higher (53.8%, 28/52). In children (age <15 yrs), 60% (9/15) of slide positives reported fever. Among 37 slide positive adults (age >14 years), 19 (51.4%) reported fever. Among these 19 cases, nine were infected with *Plasmodium falciparum *and 10 with *Plasmodium vivax. *The prevalence of infection of both species from the village surveys was higher in children (15/623 = 2.4%) as compared to adults (37/4604 = 0.8%) (χ^2 ^= 12.8, P < 0.001). The lower part of Table 1 shows data from the Clinic where 101 patients aged >14 years were interviewed; 85.2% (86/101) reported fever. Among those, 39 were infected with *P. falciparum *and 47 with *P. vivax.*

**Table 1:** Data from village surveys and the clinic on reported fever and malaria infection.

**Slide reading**	**Fever reported**	**Fever not reported**	**Fisher's exact tests of association of fever with infection**
	**Village surveys, age <15 yrs**		
*P. falciparum *+ve	2 (3.2%)	3 (0.54%)	P = 0.082
*P. vivax *+ve	7(11.1%)	3 (0.54%)	P = 0.000007
Total slides	63	560	
	**Village surveys, age >14 yrs**		
*P. falciparum *+ve	9 (1.8%)	10 (0.24%)	P = 0.00006
*P. vivax *+ve	10 (2.0%)	8 (0.19%)	P = 0.000003
Total slides	498	4106	
	**Clinic (age >14 yrs)**		
*P. falciparum *+ve	39 (45.35%)	10 (66.67%)	χ^2 ^= 0.39, P = 0.53
*P. vivax *+ve	47 (54.65%)	5 (33.33%)	χ^2 ^= 0.14, P = 0.51
Total slides	86	15	

As shown in Table 2, at least one mosquito became infected with oocysts in the batches fed on 10/28 people from the village infected with either species (35.7%). The proportion of individual patients from the Clinic who infected at least one mosquito was 43.4% (40/92). The difference between these two rates was not significant (χ^2 ^= 0.26, p = 0.6). The probability of infection of mosquitoes from people infected with *P. falciparum *was significantly less than from people infected with *P. vivax *(24.7% versus 57.1%, χ^2 ^= 11.6, P < 0.001). 77.1% (44/57) of *P. falciparum *cases reported fever. Among those, only 27.3% could infect mosquitoes. For *P. vivax *84.1% (53/63) reported fever; among those 56.6% could infect mosquitoes. More than half of the infections from whose blood no mosquitoes developed oocysts had parasite density > 3,960/μl. There was no association of probability of mosquito infection with parasite density (χ^2 ^= 1.3, P > 0.05).

**Table 2:** Results of feeding mosquitoes on human blood

	No. people from whose blood some mosquitoes developed oocysts	No. people from whose blood no mosquitoes developed oocysts	Significance of differences
	***P. falciparum***		
Villages	3 (23.1%)	10 (76.9%)	Fisher not sig.
Clinic	11 (25.0%)	33 (75.0%)	

Observable gametocytes	6 (46.2%)	7 (53.8%)	Fisher P = 0.06
No observable gametocytes	8 (18.2%)	36 (81.8%)	

Fever reported	12 (27.3%)	32 (72.7%)	Fisher P = 0.32
Fever not reported	2 (15.4%)	11 (84.6%)	

	***P. vivax***		
Villages	7 (46.7%)	8 (53.3%)	χ^2 ^= 0.41, P = 0.52
Clinic	29 (60.4%)	19 (39.6%)	

Observable gametocytes	31 (68.9%)	14 (31.1%)	χ^2 ^= 7.3, P = 0.007
No observable gametocytes	5 (27.8%)	13 (72.2%)	

Fever reported	30 (56.6%)	23 (43.4%)	Fisher P = 0.56
Fever not reported	6 (60%)	4 (40%)	

	**Parasite density (*P. falciparum *and *P. vivax*)**		
<3961/μl	21 (35.9%)	38 (64.4%)	χ^2 ^= 1.30, P = 0.25
>3960/μl	29 (47.5%)	32 (52.5%)	

Considering each species separately, the probability of infectiousness to mosquitoes was significantly related to being observably gametocyte positive in *P. vivax *(P= 0.007), but the relationship was only of borderline significance for *P. falciparum *(Fisher's exact test, P* = *0.06). Approximately 50% (7/13) of the *P. falciparum *cases with observable gametocytes failed to infect mosquitoes and 30% (14/45) of the *P. vivax *cases with observable gametocytes failed to infect. However, the difference was not significant (Fisher's exact test, P= 0.19). Approximately 21% (13/62) with no observable gametocyte of either species could infect mosquitoes.

58 of the infections with either species had observable gametocytes (11 cases from the village survey and 47 from the Clinic). Feeds on 37 of these 58 gametocyte carriers led to oocyst production. The regression of percent of mosquitoes infected on the natural log of gametocyte density was not significant (*t* = 0.87, df= 36, P= 0.39). Similarly, there was not a significant association of mean oocyst load per infected mosquito on the natural log of gametocyte density (*t* = 0.87, df = 36, P= 0.38).

## Discussion

The results from the experiments with mosquitoes showed the infectiousness of subjects from the village surveys as well as from the Clinic. These indicated that some symptomatic and asymptomatic infections of each species could infect mosquitoes. The differences in percentages of infection in different studies [2,4,14] might be explained by several possible factors. First, the method of feeding: a recent study comparing the infectivity of gametocyte carriers to mosquitoes, using membrane and direct feeding, found significantly higher proportions of mosquitoes infected and higher oocyst burdens in mosquitoes fed directly on the skin [15]. Conversely, Vanderberg [16] reported that infectivity in mosquitoes fed through a membrane usually equaled or exceeded infections by direct methods. However, most studies gave better results for direct feeding than membrane feeding. Thus studies such as the present one using direct feeding may provide a more reliable estimate of the infectious reservoir. Second, recruited subjects: in several studies mosquitoes were fed on individuals selected randomly and not on the basis of gametocytaemia [5,10,14]. But in other studies mosquitoes were fed on selected gametocyte carriers [4,17] or parasitaemic cases with or without gametocytes (as in the present study).

Another relevant factor is variability in mosquito populations [18,19], such as mosquito size [20-22]. The number of blood meals may also affect the infection rate [8,23]. A careful comparison of *Anopheles dirus*, *An. minimus *and *Anopheles maculatus *infectivity in relation to size and blood-feeding behaviour would be of interest. Moreover, variability in susceptibility between different mosquito colonies is possible [4,24].

The results in the present study showed that some cases with undetectable gametocytes could infect mosquitoes. This apparent anomaly is presumably at least partly due to larger volumes of blood in mosquito bloodmeals than are observed on slides, so that sufficient gametocytes to infect a mosquito may have been below the level of detection on blood slides. An attempt was made to see if gametocyte densities are higher in bloodmeals than finger pricks. However, it was found that gametocyte density was not significantly higher (and actually appeared to be lower) in the blood taken up by mosquitoes than in the blood from finger pricks. In some cases high densities of gametocytes were not infectious and similar results have been reported in most studies assessing human malarial infectivity to mosquitoes [3,4,11,24,25]. It has been suggested that the prevalence of gametocyte carriers is not a good indication of the infectiousness of a population to mosquitoes [10,25-27].

It is clear that, as the Ethical committee only authorized us to request people over 14 years of age to take part in our experiments, this excluded a sector of the reservoir population, the children, from the study. It is also clear that the occurrence of transmission-blocking immunity and prior histories of the subjects taking anti-malarial drugs might have influenced whether they infected mosquitoes. No data on these factors was collected and so no comment can be made on their possible influence on infectivity of people's blood to mosquitoes. However, the fact that availability of anti-malarial drugs is much more tightly controlled in Thailand than in most malarious countries should be emphasized.

**Figure 1:**
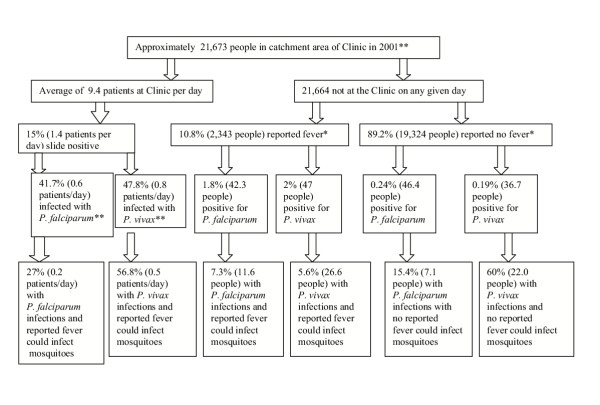
Diagram to show location of the reservoirs of infection (* data from the surveys, ** data from the Vector-borne Disease Control Clinic No. 8, Mae Hong Son Province)

From the results of the study, an attempt can be made to estimate the number of adults in the catchment area of the Clinic who were reservoirs of infection. On the basis of the number of patients visiting the Clinic per day and the catchment population from which the patients come (Figure 1), it was concluded that the main reservoir of infection for mosquitoes was not in adult patients feeling ill enough to be motivated to come to the Clinic. Among villagers, occurrence of fever is a strong indicator of likely malarial infection (Table 1). Thus, fever is an indicator of likelihood of being part of the infectious reservoir for mosquitoes. However, because there are far fewer people who are feverish than those who are not, the numbers of people in the infectious reservoir who are, or are not, feverish do not differ greatly (Fig. 1). The calculations in Figure 1 do not take into account the fact that the infected people found in the villages will remain infected for several days, whereas a new group of about nine people go to the Clinic every day. It would be useful to know for how many days people remain infectious to mosquitoes, but ethically one cannot leave detected infections untreated in order to test this. The data from Figure 1 indicate that directing an anti-gametocyte drug only to the clinic patients would be ineffective. Instead the drug would have to be targeted at the village populations after mass surveys for parasitaemia. Feverishness would assist to some extent in helping to identify people most likely to be infected. In view of the current interest in anti-gametocyte drugs [28-30], these data may be of use in deciding how such drugs would have to be targeted to have an impact on transmission.

## Authors' contributions

Aree Pethleart led the data collection team in the field and laboratory, analysed data and drafted the paper;

Somsak Prajakwong provided research facilities;

Wannapa Suwonkerd organized mosquito rearing and dissection;

Boontawee Corthong was in charge of Clinic data and provided facilities for village surveys;

Roger Webber was responsible for early planning of the work; and

Christopher Curtis supervised data analysis and edited the draft.

## References

[B1] Muirhead-Thomson R (1954). Factors determining the true reservoir of infection of *Plasmodium falciparum *and *Wuchereria bancroffi *in a West African village. Trans R Soc Trop Med Hyg.

[B2] Graves PM, Burkot TR, Carter R, Cattani JA, Lagog M, Parker J, Brabin BJ, Gibson FD, Bradley DJ, Alpers MP (1988). Measurement of malarial infectivity of human populations to mosquitoes in the Madang area, Papua, New Guinea. Parasitology.

[B3] Gamage Mendis AC, Rajakaruna J, Carter R, Mendis KN (1991). Infectious reservoir of *Plasmodium vivax *and *Plasmodium falciparum *malaria in an endemic region of Sri Lanka. Am J Trop Med Hyg.

[B4] Sattabongkot J, Maneechai N, Rosenberg R (1991). *Plasmodium vivax*: gametocyte infectivity of naturally infected Thai adults. Parasitology.

[B5] Githeko AK, Brandling Bennett AD, Beier M, Atieli F, Owaga M, Collins FH (1992). The reservoir of *Plasmodium falciparum *malaria in a holoendemic area of western Kenya. Trans R Soc Trop Med Hyg.

[B6] Rutledge LC, Ward RA, Gould DJ (1964). Studies in the feeding response of mosquitoes to nutritive solution in a new membrane feeder. Mosquito News.

[B7] Graves PM (1980). Studies on the use of a membrane feeding technique for infecting *Anopheles gambiae *with *Plasmodium falciparum.*. Trans R Soc Trop Med Hyg.

[B8] Ponnudurai T, Lensen AH, van Gemert GJ, Bensink MP, Bolmer M, Meuwissen JH (1989). Infectivity of cultured *Plasmodium falciparum *gametocytes to mosquitoes. Parasitology.

[B9] Boudin C, Lyannaz J, Bosseno MF, Carnevale P, Ambroise-Thomas P (1991). Epidemiology of *Plasmodium falciparum *in a rice field and a savanna area in Burkina Faso: seasonal fluctuations of gametocytaemia and malarial infectivity. Ann Trop Med Parasitol.

[B10] Boudin C, Olivier M, Molez JF, Chiron JP, Ambroise-Thomas P (1993). High human malarial infectivity to laboratory-bred *Anopheles gambiae *in a village in Burkina Faso. Am J Trop Med Hyg.

[B11] Tchuinkam T, Mulder B, Dechering K, Stoffels H, Verhave JP, Cot M, Carnevale P, Meuwissen JH, Robert V (1993). Experimental infections of *Anopheles gambiae *with *Plasmodium falciparum *of naturally infected gametocyte carriers in Cameroon: factors influencing the infectivity to mosquitoes. Trop Med Parasitol.

[B12] Robert V, Read AF, Essong J, Tchuinkam T, Mulder B, Verhave JP, Carnevale P (1996). Effect of gametocyte sex ratio on infectivity of *Plasmodium falciparum *to *Anopheles gambiae*. Trans R Soc Trop Med Hyg.

[B13] Gouagna LC, Mulder B, Noubissi E, Tchuinkam T, Verhave JP, Boudin C (1998). The early sporogonic cycle of *Plasmodium falciparum *in laboratory – infected *Anopheles gambiae*: an estimation of parasite efficacy. Trop Med Int Health.

[B14] Muirhead-Thomson R (1957). The malaria infectivity of an African village population to mosquitoes *(Anopheles gambiae).*. Am J Trop Med Hyg.

[B15] Bonnet S, Gouagna C, Safeukui I, Meunier JY, Boudin C (2000). Comparison of artificial membrane feeding with direct skin feeding to estimate infectiousness of *Plasmodium falciparum *gametocyte carriers to mosquitoes. Trans R Soc Trop Med Hyg.

[B16] Vanderberg J, Gwadz RW, Kreier J (1980). The transmission by mosquitoes of *Plasmodia *in the laboratory. Malaria.

[B17] Touré YT, Doumbo O, Touré A, Bagayoko M, Diallo M, Dolo A, Vernick KD, Keister DB, Muratova O, Kaslow DC (1998). Gametocyte infectivity by direct mosquito feeds in an area of seasonal malaria transmission: implications for Bancoumana, Mali as a transmission-blocking vaccine site. Am J Trop Med Hyg.

[B18] Dye C, Hasibeder G (1986). Population dynamics of mosquito-borne disease: effects of flies which bite some people more frequently than others. Trans R Soc Trop Med Hyg.

[B19] Koella JC, Lyimo EO (1996). Variability in the relationship between weight and wing length of *Anopheles gambiae *(Diptera: Culicidae). J Med Entomol.

[B20] Kitthawee S, Edman JD, Upatham ES (1992). Relationship between female *Anopheles dirus *(Diptera: Culicidae) body size and parity in a biting population. J Med Entomol.

[B21] Lyimo EO, Koella JC (1992). Relationship between body size of adult *Anopheles gambiae s.l. *and infection with the malaria parasite *Plasmodium falciparum.*. Parasitology.

[B22] Kelly R, Edman JD (1992). Mosquito size and multiple transmission of avian malaria in the laboratory. J Am Mosq Contr Assoc.

[B23] Hurd H, Hogg H, Renshaw M (1995). Interaction between bloodfeeding, fecundity and infection in mosquitoes. Parasit Today.

[B24] Rutledge LC, Gould DJ, Tantichareon B (1969). Factors affecting the infection of anophelines with human malaria in Thailand. Trans R Soc Trop Med Hyg.

[B25] Jeffery GM, Eyles DE (1955). Infectivity to mosquitoes of *Plasmodium falciparum *as related to gametocyte density and duration of infection. Am J Trop Me Hyg.

[B26] Carter R, Gwadz RW (1980). Infectiousness and gamete immunization in malaria.

[B27] Carter R, Graves PM (1988). Malaria: Principles and Practice of Malariology.

[B28] Chavalitshewinkoon-Petmitr P, Pongvilairat G, Auparakkitanon S, Wilairat P (2000). Gametocytocidal activity of pyronaridine and DNA topoisomerase II inhibitors against multidrug-resistant *Plasmodium falciparum *in vitro. Parasitol Int.

[B29] van Vugt M, Looareesuwan S, Wilairatana P, McGready R, Villegas L, Gathmann I, Mull R, Brockman A, White NJ, Nosten F (2000). Artemether-lumefantrine for the treatment of multidrug-resistant falciparum malaria. Trans R Soc Trop Med Hyg.

[B30] Targett G, Drakeley C, Jawara M, von Seidlein L, Coleman R, Deen J, Pinder M, Doherty T, Sutherland C, Walraven G, Milligan P (2001). Artesunate reduces, but does not prevent, post-treatment transmission of *Plasmodium falciparum *to *Anopheles gambiae.*. J Infect Dis.

